# Olfactory Attraction of the Hornet *Vespa velutina* to Honeybee Colony Odors and Pheromones

**DOI:** 10.1371/journal.pone.0115943

**Published:** 2014-12-30

**Authors:** Antoine Couto, Karine Monceau, Olivier Bonnard, Denis Thiéry, Jean-Christophe Sandoz

**Affiliations:** 1 Evolution Genomes and Speciation Lab (LEGS), CNRS UPR 9034, Gif-sur-Yvette, France; 2 Equipe Ecologie Evolutive, UMR CNRS 6282 Biogéosciences, Université de Bourgogne, Dijon, France; 3 UMR 1065 Santé et Agroécologie du Vignoble, INRA, Villenave d’Ornon, France; 4 Université de Bordeaux, ISVV, UMR 1065 Santé et Agroécologie du Vignoble, Bordeaux Sciences Agro, Villenave d’Ornon, France; University of Arizona, United States of America

## Abstract

Since the beginning of the last century, the number of biological invasions has continuously increased worldwide. Due to their environmental and economical consequences, invasive species are now a major concern. Social wasps are particularly efficient invaders because of their distinctive biology and behavior. Among them, the yellow-legged hornet, *Vespa velutina*, is a keen hunter of domestic honeybees. Its recent introduction to Europe may induce important beekeeping, pollination, and biodiversity problems. Hornets use olfactory cues for the long-range detection of food sources, in this case the location of honeybee colonies, but the exact nature of these cues remains unknown. Here, we studied the orientation behavior of *V. velutina* workers towards a range of hive products and protein sources, as well as towards prominent chemical substances emitted by these food sources. In a multiple choice test performed under controlled laboratory conditions, we found that hornets are strongly attracted to the odor of some hive products, especially pollen and honey. When testing specific compounds, the honeybee aggregation pheromone, geraniol, proved highly attractive. Pheromones produced by honeybee larvae or by the queen were also of interest to hornet workers, albeit to a lesser extent. Our results indicate that *V. velutina* workers are selectively attracted towards olfactory cues from hives (stored food, brood, and queen), which may signal a high prey density. This study opens new perspectives for understanding hornets’ hunting behavior and paves the way for developing efficient trapping strategies against this invasive species.

## Introduction

Since the beginning of the 20^th^ century, as a result of globalization and the development of international exchanges, biological invasions have critically increased [1]–[4]. Invasive species frequently disturb biodiversity by competing with indigenous species for habitat and food resources and/or by exerting a strong predation pressure on local species. Due to the ecological and economical problems that ensue from biological invasions, they have become a major concern worldwide [5]–[6]. Introduced species do not however all become invasive and only ∼1% actually lead to permanent invasions [7]. Social insects, like social wasps, are particularly efficient invaders. They live in populous colonies organized according to highly efficient task allocation systems, which confers them outstanding abilities for exploiting local food resources and outcompeting local species [8]. Social wasps gather food resources from the surroundings of the nest, mostly gathering carbohydrates and proteins [9]. These generalist hunters typically find protein resources by preying on numerous insect species including many pollinators. Because of the current decline of pollinator populations [10]–[11] and of the severe ecological consequences of the introduction of such generalist hunters in new ecosystems [12], acquiring a better knowledge of the predation behavior of social wasps has become a crucial endeavor.

Predation site selection by social wasps is strongly influenced by prey density [13]–[14], as a high number of preys at a particular location gives rise to conspicuous visual and olfactory cues. Social wasps are keen learners which are able to associate color or odor cues with food reward [15]–[16]. Thus, after several hunting bouts on a particular hunting site, these insects learn the cues that allow them to localize their prey [9], [17]–[21]. This high behavioral plasticity supported by excellent cognitive abilities has been proposed as another explanation for the invasive success of social wasps [22]–[24].

The yellow-legged hornet, *Vespa velutina*, is an attractive species for studying hornets’ hunting behavior. Recently, this hornet originating from China has been accidently introduced to new locations in Western Europe and in Korea [25]–[27]. In Europe, this species was first observed in 2004 [27]–[28]. Like other wasps of the Vespinae subfamily, hornets prey on various arthropod species but also scavenge on dead vertebrate flesh (fish and meat) [9], [29]–[32]. Above all, however, *V. velutina* hunts domestic honeybees, including the Asian honeybee, *Apis cerana* (in their natural range) [33] and the European honeybee, *Apis mellifera* (both in their natural and introduction ranges) [34]. Because of the strong and immediate impact of this hornet’s hunting pressure on honeybee colonies, several studies have been dedicated to honeybees’ defense behavior against it. These studies essentially showed that most European honeybees are not able to display efficient anti-predator behaviors contrary to Asian honeybees which coevolved with several bee-hunting hornet species [35]–[42]. In invaded areas, hornets’ feeding sites are thus primarily apiaries, because they present an abundant and defenseless prey source. However, little is known about the hunting behavior of *V. velutina* [43]–[44] and how they locate and choose these feeding sites.

At the peak of predation, as many as 20 hornets can be seen hovering simultaneously in front of a given hive, catching honeybee foragers on the fly. This hunting strategy strongly impairs the foraging efficiency of bees [43]. Two mechanisms may be involved in such high numbers of preying hornets. First, *V. velutina* workers could learn the characteristics and/or location of feeding sites, as the same foragers can be seen several times hunting on the same apiary [44]. Second, like other Vespine wasps, hornets may recruit nestmates to feeding sites [35], [45]. In any case, honeybee colonies are especially attractive to hornets, but the exact cues they may use to locate honeybee colonies in the first place remain unclear. Recently, a laboratory study showed that *V. velutina* workers can use both visual and olfactory cues to locate bees [46]. In the wild, visual cues are critical at short-range for catching flying honeybee foragers, while olfactory cues may be more relevant for long-range attraction to honeybees hives. To this day such odorants remain unknown despite their considerable interest for understanding *V. velutina* foraging tactic. In theory, predators can take advantage of different categories of olfactory cues [47]. The most immediate and accurate way to locate a prey is to use olfactory cues emanating directly from the prey (kairomones) [48]–[50]. However, if such odorants are not sufficient for guiding the predator, other strategies using indirect olfactory cues which emanate from the habitat or from the food of the prey have been reported [51]–[54]. Hence, hornets may be attracted to volatiles emitted by honeybees, to volatiles emitted by the bee hives as well as to odorants emitted by the flesh they sometimes scavenge on. In the present study, we tested the attraction behavior of *V. velutina* workers to olfactory stimuli in a multiple choice test in controlled conditions. We first asked which elements (hive products, honeybees or fish or meat samples) may attract hornets. Then, we tested hornets’ relative attraction to a range of different molecules that are typical honeybee- or flesh-emitted volatiles. In particular, we tested a number of known honeybee pheromone that may act as kairomones for hornets.

## Materials and Methods

### Animal collection and caging procedure

**Figure 1:**
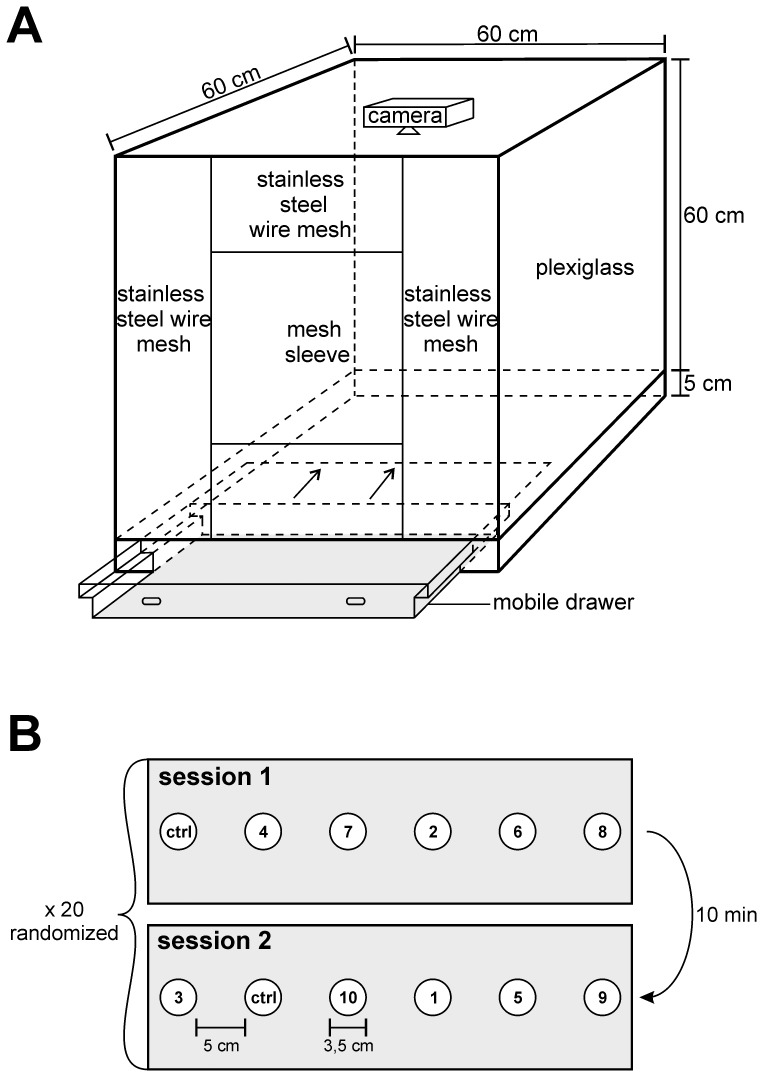
Experimental setup. **A.** Schematic drawing of the cubic wooden cage (60×60×60 cm) used for the experiments. The front part was equipped with a mesh sleeve for introducing hornets into the cage. The base contained a 5 cm deep mobile drawer (in grey) and a stainless steel plate used to securely deliver tested stimuli. Inside the cage and attached to the roof, a digital camcorder allowed the recording of hornets’ behavior. **B.** Experimental procedure used in the first experiment. Each of the 20 replications was divided in 2 consecutive sessions separated by a 10 minute interval. Within a session, 5 stimuli among the 10 tested (numbered circles) were placed 5 cm apart in the mobile drawer, and delivered together with a control (ctrl circle). The sequence of stimuli presentation and exact location in the drawer were fully randomized. The second experiment was similarly designed but contained 4 sessions of 5 stimuli among the 20 tested.

*Vespa velutina* workers preying on honeybee hives were individually caught with a butterfly net between August and November 2013, at the experimental apiary of INRA Bordeaux-Aquitaine, France (GPS: N44°47′27.05″ W0°34′38.35″). Animals were then brought to a dark and temperature-controlled (22°C) experimental room in the laboratory containing a cubic cage (60×60×60 cm, described in [55]). Groups of 10 hornets were transferred to the cage and were allowed to move freely for 3 hours during a period of acclimatization prior to the experiments. The base of the cage contained a mobile drawer with a stainless steel plate allowing to securely deliver stimuli into the cage (Fig. 1A). A digital camcorder (HD pro webcam C920; Logitech, Lausanne, Switzerland) was placed inside the cage and attached to its roof to record *V. velutina* behavior during the tests. The cage was illuminated with red light by means of an incandescent light bulb. Red light allowed filming the hornets while precluding their use of visual cues during the experiments, since, as shown for the closely related species *V. crabro,* hornets lack red-light sensitive photoreceptors [56]. The Virtualdub software (http://www.virtualdub.org) was used for recording high quality video at a frame rate of 22 fps.

### Experiment 1

In order to screen attractive food sources or hive products, 10 different stimuli were used. For all stimuli, the same mass of substance was presented, i.e. 80 mg±2 mg, as it equates to the average weight of a honeybee worker. The stimuli were:

*- Honey*: organic raw mixed-flower honey (not warmed), obtained from a local beekeeper

*- Pollen*: organic, bee-collected, fresh pollen (not dried), obtained from a local beekeeper

*- Wax*: new honeybee comb foundation wax obtained from a local beekeeping shop

*- Propolis*: purified in ethanol (ethanol was evaporated after filtration in liquid phase)

*- Bee boost*: commercial synthetic honeybee queen mandibular pheromone (QMP) on a plastic strip (Contech, Victoria, BC, Canada). QMP is a blend of five constituents, 9-oxo-(E)-2-decenoic acid (9-ODA), two enantiomers of its biosynthetic precursor, (R)- and (S)-9-hydroxy-(E)-2-decenoic acid (9-HDA) and two other compounds, methyl p-hydroxybenzoate (HOB) and 4-hydroxy-3-methoxyphenylethanol (HVA) [57].

*- Honeybee*: worker honeybee taken from a hornet-predated hive

*- Larva*: honeybee larva taken from a hornet-predated hive

*- Meat*: minced beef meat, obtained from a local supermarket

*- Fish*: salmon flesh, obtained from a local supermarket

*- Wet paper*: white cellulose wadding moisturized with distilled water

Stimuli were individually placed in Ø 35 mm Petri dishes (Petri dishes, EL46.1; Roth, Lauterbourg, France) and delivered into the cage using the mobile drawer. Six Petri dishes were placed at a time in the drawer, and were spaced 5 cm apart. Hornets were subjected to 2 consecutive sessions separated by a 10 minute interval (Fig. 1B). Each session presented 5 of the 10 stimuli plus a joint control (empty Petri dish). The order of presentation of the stimuli (session 1 or 2), as well as their location in the drawer, were fully randomized using Research Randomizer (http://www.randomizer.org). Before each session, the mobile drawer was thoroughly washed with ethanol to remove any stimulus residues. Following introduction of the Petri dishes, hornets’ behavior in the drawer area was recorded for 15 minutes with the digital camcorder. This experiment was repeated 20 times (20×2 sessions) with different groups of 10 hornets. New stimuli were used for each test.

### Experiment 2

Food odors are complex blends of odorant molecules present in variable ratios. To attempt to isolate attractive odorants for hornets, we selected 20 volatile compounds (Sigma-Aldrich, Steinheim, Germany) based on their occurrence at high concentration in the different food sources of *V. velutina*. Table 1 shows the list of tested compounds. For commodity, the tested stimuli were categorized in 3 main groups (honeybee, seafood and meat odors), although each stimulus can also be emitted by a variety of other sources. For each odorant, a filter paper piece (1 cm^2^; Whatman, Maidstone, England) was soaked with 5 µL of the odor solution and placed in a Ø 35 mm Petri dish. Liquid substances were presented pure, while odorants in solid form at room temperature (homovanillyl alcohol, 10-hydroxydecanoic acid, methyl 4-hydroxybenzoate, methyl palmitate and ethyl oleate) were dissolved in 2-propanol and presented at a dose of 50 µg/µL. 2-propanol was allowed to evaporate for 1 min before presenting the stimuli. As in experiment 1, 5 stimuli and a control (1 cm^2^ filter paper without a stimulus) were delivered at a time into the cage using the mobile drawer. The 20 stimuli were thus delivered in 4 consecutive sessions lasting 15 minutes each and separated by a 10 minute interval. To remove residual odors before each session, the drawer was thoroughly washed with ethanol. Sequence of stimulus presentation and location in the drawer were randomized as above. The experiment was repeated 20 times (20×4 sessions) on different groups of 10 hornets. New stimuli were used for each test.

**Table 1:** List of molecules tested in Experiment 2.

Chemical name	CAS	MW (g.mol^-1^)	Purity	Sources	References
isopentyl acetate	123-92-2	130.18	99%	**bee alarm pheromone**	[62], [76]
2-heptanone	110-43-0	114.18	99%	**bee alarm pheromone**	[62], [77]–[78]
geraniol	106-24-1	154.24	98%	**bee aggregation pheromone**	[61], [62]–[63]
citral	5392-40-5	152.23	95%	**bee aggregation pheromone**	[79]–[80]
homovanillyl alcohol	2380-78-1	168.18	99%	**bee queen pheromone**	[57]–[81]
10-hydroxydecanoic acid	1679-53-4	188.26	99%	**bee queen pheromone**	[82]–[83]
methyl 4-hydroxybenzoate	99-76-3	152.14	99%	**bee queen pheromone**	[84]
methyl palmitate	112-39-0	270.45	99%	**bee brood pheromone**	[85]
ethyl oleate	111-62-6	310.51	98%	**bee brood pheromone**	[86]
β-ocimene (E, Z mixture)	13877-91-3	136.23	90%	**bee brood pheromone**	[65]
heptanal	111-71-7	114.18	95%	**seafood**/plants/meat	[67], [87]–[89]
(E)-2-nonenal	18829-56-6	140.22	95%	**seafood/**aged beer	[67]–[68], [87], [90]
(E, E)-2,4-octadienal	5577-44-6	124.18	95%	**Seafood**	[67], [91]
(E, Z)-2,6-nonadienal	557-48-2	138.20	96%	**seafood/**meat/plants	[67]–[68], [87], [89], [92]–[93]
p-xylene	106-42-3	106.16	99%	**seafood/**meat/plants	[67]–[69], [89]
1-octen-3ol	3391-86-4	128.21	98%	**meat/**honey	[67]–[68], [87], [89], [94]–[95]
benzaldehyde	100-52-7	106.12	98%	**meat/**plant	[88]–[89], [94], [96]–[97]
octanoic acid	124-07-2	144.21	98%	**meat/**plant	[88], [97]
4-ethyloctanoic acid	16493-80-4	172.26	98%	**meat**	[98]
(E, E)-2,4-decadienal	25152-84-5	152.23	89%	**meat/**plant/seafood	[67], [87], [92], [94], [96], [99]

The table presents the chemical names of the 20 molecules tested in Experiment 2. CAS number, molecular weight and purity are indicated in the second, third and fourth column respectively. The fifth column shows a non exhaustive list of sources that emit these molecules, as mentioned in the literature cited in the last column.

### Videos analysis

Preliminary observations of hornet’s behavior showed that in Experiment 1, some food sources could be consumed within 10 minutes of the presentation of the stimuli. Thus, in this experiment, hornet behavior was only analyzed during the first 10 minutes of each recording. In Experiment 2, none of the odorant sources were consumed, so hornet behavior was analyzed during the full 15 min of the experiment. Videos were visualized using Movie Maker V. 2011 software (Microsoft Corporation, Redmond, USA) allowing us to precisely record the number of visits and the amount of time hornets spent on each stimulus. At each separate occasion a hornet entered a Petri dish (at least with its entire head), times of entry and of exit were recorded. Hornet attraction to the different stimuli was measured according to two variables: 1) *Visit number*: the total number of hornet visits during each session; and 2) *Total visit duration*: the sum of all individual visit durations performed by hornets during this session.

### Statistics

A Friedman test was used to compare visit frequencies and durations on the control stimuli during different sessions. To evaluate the attractiveness of the different stimuli, we compared the number of visits to each test stimulus with its joint control (i.e. with all control stimuli presented in the same sessions), using a Wilcoxon paired sample test. Likewise, the amount of time that hornets spent on each stimulus was compared to its joint control with a Wilcoxon paired sample test. As multiple comparisons were carried out with the same control (always 5 stimuli with 1 control), we used a Bonferroni correction to reduce type I errors (α_corrected_  = 0.05/5 = 0.01). A p value below α_corrected_ was considered as statistically significant, while a value between α_corrected_ and the standard 0.05 threshold was only regarded as near-significant (but potentially biologically relevant). Significance levels are presented in the figures as follows: ***: p<0.0001; **: p<0.001; *: p<0.01; (*) 0.01<p<0.05).

## Results

The behavior of 400 hornets was observed in two experiments aiming to test their olfactory attraction towards food sources (Experiment 1) or individual odorants (Experiment 2).

### Experiment 1

In the first experiment, *V. velutina* workers were presented with several food sources and different elements of a honeybee colony. The hornets could explore the drawer at the base of the cage, containing 6 Petri dishes presenting 5 stimuli and a control. Because hornets were free to move around the cage, visits to the stimuli may occasionally occur through random exploration. Visits to control stimuli therefore allows us to evaluate the intensity of hornets’ exploration behavior. Stimulus exploration was similar between the first and second session, as quantified through the number of visits to the control (Wilcoxon test, Z  = 1.24, p  = 0.21, NS).

**Figure 2:**
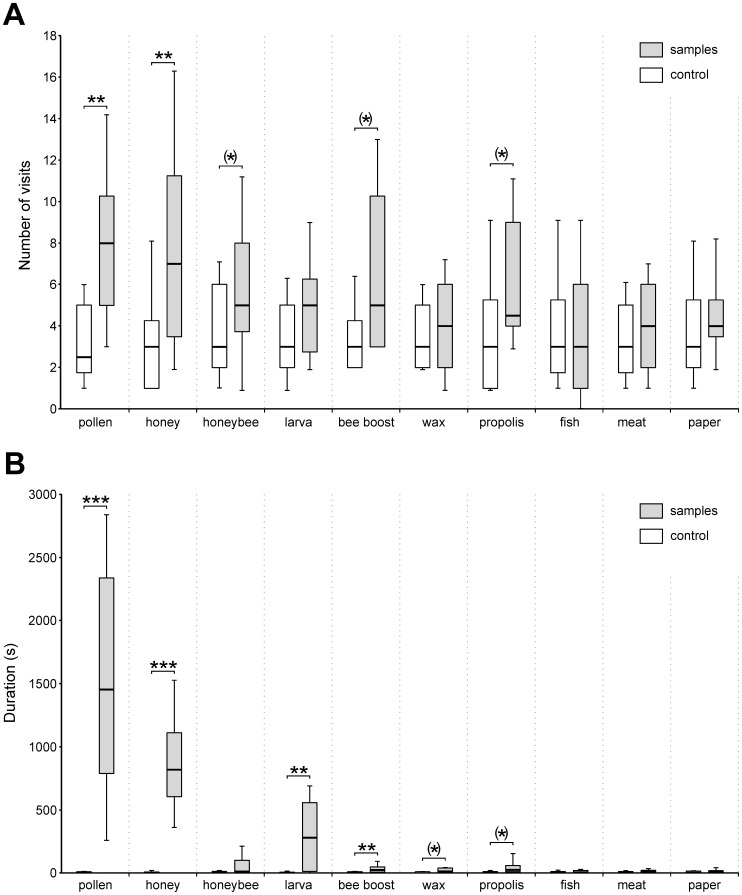
Hornet attraction towards hive products and other food samples. **A.** Distribution of the number of visits on each sample for groups of 10 hornets. Bold lines show the median number of visits per session (20 replications, N  = 200 hornets). The boxes represent the first and third quartiles (25–75%), while whiskers represent the distribution from 10 to 90%. The thick vertical dashed lines separate the different sources emitting these molecules (from left to right: honeybee, fish, meat). **B.** Distribution of the cumulative duration that groups of 10 hornets spent on each sample. Bold lines show the median duration by session (20 replications, N  = 200 hornets). Grey bars: samples; white bars: control. Comparison between samples and control were done with Wilcoxon tests (***: p<0.0001; **: p<0.001; *: p<0.01; (*) 0.01<p<0.05).

Visits to the control stimuli presented concomitantly with each stimulus were highly similar (Fig. 2A; median visit number between 2.5 and 3.0) and indicated that exploration behavior was homogenous in the whole experiment (Friedman test, Chi^2^ = 6.40, p  = 0.70, NS, 9 df). When evaluating the attractiveness of the different stimuli, we observed that hornets visited *honey* and *pollen* significantly more often than the control stimulus (Wilcoxon test, Z  = 3.33, p<0.001 and Z  = 3.57, p<0.001). Although hornets showed no significant preference for other stimuli, some showed near-significant attraction (α_corrected_  = 0.01<p<0.05): *propolis* (Wilcoxon test, Z  = 2.37, p  = 0.017), *bee boost* (Wilcoxon test, Z  = 2.48, p  = 0.013) and *honeybee* (Wilcoxon test, Z  = 2.07, p  = 0.038).

When evaluating the duration that hornets spent visiting each stimulus (Fig. 2B), we observed that they consistently spent more time on *honey* and *pollen* than on the control (Wilcoxon test, Z  = 3.92, p<0.0001 and Z  = 3.92, p<0.0001, respectively). In addition, the duration spent on *larva* and *bee boost* was also significantly longer (Wilcoxon test, Z  = 3.82, p<0.001 and Z  = 3.32, p<0.001) than the control. The time hornets spent visiting *propolis* (Wilcoxon test, Z  = 2.39, p  = 0.017) and *wax* (Wilcoxon test, Z  = 2.12, p  = 0.033) was only near-significantly longer than on the control (α_corrected_ <p<0.05).

### Experiment 2

**Figure 3:**
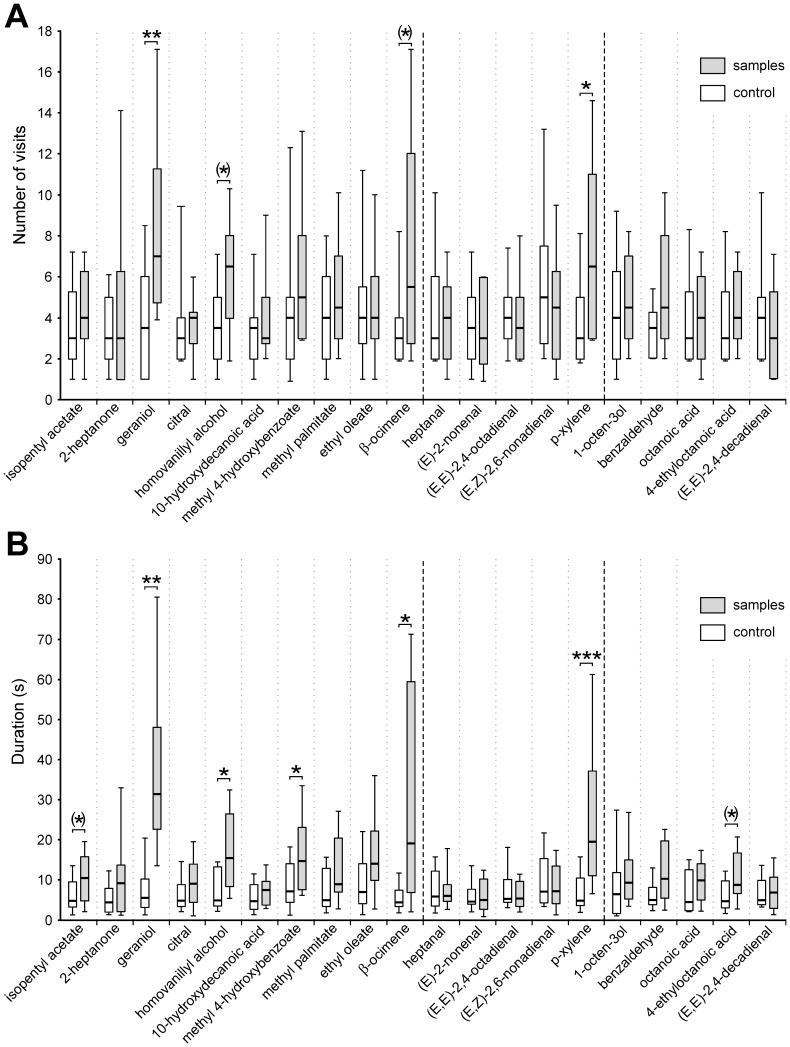
Hornet attraction towards chemical substances. **A.** Distribution of the number of visits on each stimulus exhibited by groups of 10 hornets. Bold lines show the median number of visits per session (20 replications, N  = 200 hornets). The boxes represent the first and third quartiles, while whiskers represent the distribution from 10 to 90%. **B.** Distribution of the cumulative duration that groups of 10 hornets spent on each stimulus. Bold lines show the median duration by session (20 replications, N  = 200 hornets). Grey bars: stimuli; white bars: control. Comparison between odor stimuli and control were done with Wilcoxon tests (***: p<0.0001; **: p<0.001; *: p<0.01; (*) 0.01<p<0.05).

In the second experiment, we asked which food odorants or honeybee pheromones may attract *V. velutina* (Table 1). As in experiment 1, hornets explored the newly accessible drawer and visited the stimuli (Fig. 3A). When comparing visits to the control stimuli, we did not find any significant variation among the four sessions, although a trend for increased exploration between sessions 1 and 4 appeared (Friedman test, Chi^2^ = 7.77, p  = 0.051, NS, 3 df). In any case, full randomization of stimulus sequence presentation allowed avoiding any influence of exploration changes on the results. Visits to control stimuli presented concomitantly with each stimulus were homogeneous (Fig. 3A, Median visit number between 3.0 and 5.0, Friedman test, Chi^2^ = 14.27, p  = 0.077, NS, 19 df).

When evaluating the attractiveness of single odorant stimuli, we observed more visits on *geraniol* and *p-xylene* than on the control (Wilcoxon test, Z  = 3.64, p<0.001 and Z  = 3.07, p<0.01). Although hornets showed no significant preference for other stimuli, *homovanillyl alcohol* (Wilcoxon test, Z  = 2.22, p  = 0.026) and *β-ocimene* (Wilcoxon test, Z  = 2.12, p  = 0.034) showed near-significant attraction (α_corrected_ <p<0.05).

Consistently with visit numbers, visit duration measurements showed that hornets spent more time on *geraniol* and *p-xylene* (Fig. 3B) than on the control (Wilcoxon test, Z  = 3.81, p<0.001 and Z  = 3.92, p<0.0001). Furthermore, hornets also spent longer periods of time on *homovanillyl alcohol* (Wilcoxon test, Z  = 2.69, p<0.01), *methyl 4-hydroxybenzoate* (Wilcoxon test, Z  = 2.87, p<0.01) and *β-ocimene* (Wilcoxon test, Z  = 2.95, p<0.01) than on the control. Lastly, the period of time hornets spent visiting *4-ethyloctanoic acid* (Wilcoxon test, Z  = 2.54, p  = 0.011) and *isopentyl acetate* (Wilcoxon test, Z  = 2.13, p  = 0.033) was near-significantly longer than on the control (α_corrected_ <p<0.05).

## Discussion

Using a controlled laboratory assay to measure hornets’ olfactory attraction, we found that they are primarily attracted to (and spent more time on) two products from honeybee hives, *pollen* and *honey*. By contrast, the honeybees themselves were only near-significantly attractive. Among individual odorants, however, one honeybee aggregation pheromone, *geraniol,* induced significant attraction and longer visit durations than the control. Our experiments also allowed observing a few more subtle effects. Honeybee *larva* and *bee boost* induced longer visit durations than the control, and similar results were observed when presenting some of their compounds alone such as *β-ocimene* (larva pheromone), *homovanillyl alcohol* (HVA) or *methyl-4-hydroxybenzoate* (HOB) (queen pheromones present in the *bee boost*). Stimuli associated with protein food sources, such as fish and meat samples or typical compounds from these protein sources did not induce any particular attraction, with the exception of one compound, *p-xylene*. Thus some stimuli were attractive: *honey* and *pollen* in experiment 1 and *geraniol* and *p-xylene* in experiment 2 whereas some stimuli only induced longer visit durations: *larvae* and *bee boost* in experiment 1 and *homovanillyl alcohol* and *methyl 4-hydroxybenzoate* in experiment 2.

### Removal of potential visual cues

Hornets are known to use both visual and olfactory cues to locate food sources [46]. In the present study, we aimed to compare the attractiveness of a range of olfactory cues without any influence from visual information provided by the samples or the experimental cage. Because of the need to film the hornets, our assays were conducted under low levels of red light. Intracellular recordings of photoreceptors have shown that the visible spectrum of hornets (in this case *V. crabro*) starts around 300 nm and quickly decreases around 600 nm [56] due to a lack of red photoreceptors as in many hymenopterans [58]. Thus, in this context, visual cues were, if not totally absent, at least very strongly reduced. In addition, to avoid any potential bias of each samples’ location with respect to the red light, sample positions were placed randomly at each session. We are therefore confident that hornets’ orientation behavior in our experiments was influenced by olfactory cues and not by visual cues.

### The prey itself or its products?

As many hornet species, and especially *V. velutina*, are keen honeybee hunters [33], [35], [37], [41] and the hornet workers we tested were caught while hunting on honeybee hives, we expected them to be primarily attracted to and to feed on their prey. This was not the case as the *honeybee* samples were only mildly attractive (Fig. 2A) and hornets did not remain on them when encountering them (Fig. 2B). By contrast, honeybee products, *pollen* and *honey*, were highly attractive (Fig. 2A). They strongly attracted hornets, sometimes even in groups and the hornets stopped for long periods of time on these samples, eventually feeding on them (Fig. 2B). Previous studies showed that the foraging behavior of some parasitic wasps [51], [53] and some social wasps [59]–[60], are influenced by cues associated with their prey but not emanating directly from the prey. For instance, the social wasp *Mischocyttarus flavitarsis* uses olfactory cues originating from a plant damaged by the feeding activity of its prey [60]. One important activity of honeybees during summer is to store high quantities of food within their colony and such stores may become a conspicuous signal for the predator. Taking advantage of cues emitted by hive stores could be more efficient for the hornet than searching for individual flying honeybees. We therefore hypothesize that *pollen* and *honey* odors emanating from the hives’ food stores represent prominent distant cues helping *V. velutina* foragers to find their prey.

Hornets gather proteins to feed their larvae but also need carbohydrates (and possibly proteins) for their own needs. As *V. velutina* workers were sometimes seen feeding on *pollen* and *honey* in our experiment (Fig. 2B), we cannot exclude the possibility that the long time hornets spent on these stimuli may be related to their nutritional quality. However, the significant numbers of visits recorded to these stimuli demonstrate their olfactory attractiveness for hornets. In addition, during hunting, *V. velutina* workers usually hover at the hive entrance and catch flying honeybee foragers, some of which are loaded with pollen and/or nectar [43]. Hornets have never been observed feeding directly on pollen pellets from caught workers. Therefore, we rather favor the idea that hornets’ attraction to pollen and honey odors was related to a potential role of these odors as long-distance signals for the presence of hives.

### Could honeybee pheromones be used as kairomonal signals?

There is increasing literature reporting how prey pheromone can be counterused as a kairomonal signal by predators or parasitoids [47]–[50]. In addition to the observation that hive products strongly attract *V. velutina*, our experiments revealed that hornets can also be attracted to some honeybee pheromones. The only other honeybee-related stimulus that significantly attracted hornets was the honeybee worker aggregation pheromone component, *geraniol.* Hornets chose to orient towards *geraniol* (Fig. 3A) and remained on this stimulus for long periods of time (Fig. 3B). Geraniol is a Nasanov gland secretion, which acts as an aggregation pheromone, attracting honeybee workers during swarming or at the colony entrance [61]–[63]. Because of its role in aggregating honeybees, geraniol may represent an honest signal for hornets, indicating high densities of honeybee workers. It may thus work as a kairomone, possibly together with hive products to help hornets finding honeybee colonies.

The other bee pheromone constituents tested in our work (Table 1) did not induce any significant increase in the number of visits, but some (*β-ocimene*, *homovanillyl alcohol* and *methyl-4-hydroxybenzoate)* were found to act as arrestants (Fig. 3B). Indeed, in this experiment, we noticed that visit duration increased when hornets exhibited typical arrestment responses [64] with klinotaxis behavior around the stimuli. *β*-*ocimene*, a volatile pheromone produced by honeybee larvae [65] showed only near-significant attraction but significant arrestment (Fig. 3. A, B). Consistently, *larvae*, when presented in the first experiment, did not induce any significant attraction but induced longer visit durations once hornets had found them (Fig. 2. A, B). These results suggest the existence of arrestment responses by hornets towards *β-ocimene,* which could also act as a kairomone, giving relevant cues for locating *larvae*.

A similar situation was observed for queen pheromones. While in the first experiment, the *bee boost*, an artificial blend of 5 components composing the honeybee Queen Mandibular Pheromones (QMP) showed near-significant attraction and significantly longer durations, two QMP components, *homovanillyl alcohol* (HVA) and *methyl-4-hydroxybenzoate* (HOB), showed the same pattern in experiment 2, with a significant arrestment to both stimuli. Here again, some honeybee queen pheromones may act as kairomones for hornets.

Such a result is consistent with previous studies on another hornet species, *V. bicolor*, which was shown to be attracted to (Z)-11-eicosen-1-ol, a major component in the alarm pheromone of both Asian and European honeybees [50]. Another prominent example is found in the wasp *Vespula germanica* which uses the pheromone produced by sexually active Mediterranean fruit fly males as olfactory cues to locate its prey [48]. Taken together, our results may indicate that hornets use a combination of honeybee-related odorants to locate hives from a distance (attraction to *honey*, *pollen* or *geraniol*). In addition, when a honeybee colony is weak, hornets sometimes enter the hive to plunder its resources. When in the hive, they may use particular honeybee pheromones as suggested by the significant arrestment observed to larvae, worker and queen pheromones.

### Lack of attraction to generic animal protein sources

Vespine wasps are generalist foragers that hunt arthropod preys but also collect meat from vertebrates to feed their larvae [31]–[32]. They are notorious pests in butchers’ and fishmongers’ stalls where they cut small pieces of meat and fish and fly away [9], [29]. In this study, unlike hive product, neither *meat* nor *fish* attracted hornets significantly (Fig. 2.A). As also shown by the low time spent on these stimuli (Fig. 2.B), we did not observe any hornets feeding on *meat* or *fish* samples. Thus, contrary to our expectations, hornets fed on only two stimuli considered as protein sources, mainly *pollen* and to a lesser extend *larva,* but neglected other animal proteins. Although carbonyl compounds resulting from lipid oxidation contribute strongly to the typical fishy odor [66]–[67], hornets exhibited significant attraction and arrestment responses only towards one fishy odor, *p-xylene* (Fig. 3. A, B). This compound can be a product of carotenoid degradation in fish flesh and has been reported as an active component in many seafood products [67], [68]–[69]. But, since *fish* flesh was not attractive in our experiment, the reason why *p-xylene* was of interest to *V. velutina* remains unclear. This chemical may also emanate from many different sources such as some fruit trees and some orchids [70]–[71].

### Recruitement and learning as potential mechanisms

Hornets’ attraction to particular stimuli in our experimental setup may be the product of both innate attraction and appetitive learning processes. Social insects are able to recruit nestmates to valuable food sources through active communication. For instance, honeybees can actively promote food sources to nestmates with a sophisticated dance [72]–[73]. Several foraging recruitment forms by means of scent marks have been reported in Vespoidea [35], [45], [74]. When the predation activity of *V. velutina* reaches its peak, up to 20 individuals can hunt on a single hive at the same time [43]. Therefore, the high number of hornets present at the hive entrance might be due to recruitment by conspecifics. Some of our hornets may have been caught during their first visits to the location and may have displayed innate attraction to the stimuli. However, taking into account the high learning abilities of Vespine wasps [15]–[16], [18], [20], tested individuals may also have learned hive cues in the course of preying visits to the hive. Indeed in a previous study the same individuals were observed several times hunting at the same site [44]. In the framework of associative learning, hive and honeybee odors may correspond to conditioned stimuli (CS) while the successful catch of a prey may act as reinforcement. If so, then our experiments reflect the attraction of honeybee-specialist hunters, which would explain the lack of attraction to meat/fish stimuli. Similar experiments comparing the behavior of *V. velutina* workers caught on different food sources should clarify how foraging specialization influences olfactory attraction in hornets.

### Outlook

In our experiments, *V. velutina* hornets mainly exhibited interest to hive products and to honeybee emitted pheromones but not to honeybees themselves. All these attractive stimuli seem to share a common feature: they might signal not only the presence of honeybees, but their presence at a high density. Interestingly, *pollen* was strongly attractive and the prey spectrum of *V. velutina* includes a large variety of pollinators such as social Hymenoptera and hoverflies [75]. Preliminary trapping experiments in the field gave promising results for using *pollen’s* attractiveness for protecting hives from hornet aggression. Such field experiments will now be performed on a larger scale, testing the trapping properties of the stimuli identified in the present study, alone or in combination.
